# Synthesis, Characterization, and Cytotoxicity of Some New 5-Aminopyrazole and Pyrazolo[1,5-*a*]pyrimidine Derivatives

**DOI:** 10.3797/scipharm.1409-14

**Published:** 2014-10-24

**Authors:** Ashraf S. Hassan, Taghrid S. Hafez, Souad A. Osman

**Affiliations:** Department of Organometallic and Organometalloid Chemistry, National Research Centre, El-Buhoth St, Cairo, Doki, Egypt

**Keywords:** *N*-Substituted cyanoacetamide, Ketene *N,S*-acetals, Pyrazolo[1,5-*a*]pyrimidines, 5-Aminopyrazoles, Cytotoxic activity

## Abstract

5-Amino-*N*-aryl-3-[(4-methoxyphenyl)amino]-1*H*-pyrazole-4-carboxamides **4a–c** were synthesized by the reaction of *N*-(aryl)-2-cyano-3-[(4-methoxyphenyl)amino]-3-(methylthio)acrylamides **3a–c** with hydrazine hydrate in ethanol. The reaction of 5-amino-*N*-aryl-1*H*-pyrazoles **4a–c** with acetylacetone **5** or 2-(4-methoxybenzylidene)malononitrile **8** yielded the pyrazolo[1,5-*a*]pyrimidine derivatives **7a–c** and **10a–c**, respectively. The structures of the synthesized compounds were established based on elemental analysis and spectral data (IR, MS, ^1^H-NMR, and ^13^C-NMR). Representative examples of the new synthesized products were screened for their *in vitro* cytotoxic activity against Ehrlich Ascites Carcinoma (EAC) cells.

## Introduction

The design and synthesis of novel mono-, di-, and polycyclic fused nitrogen heterocyclic compounds is among the active principles in chemical materials, particularly those displaying strategic roles in the development of different industries, especially from the biological point of view. Pyrazoles and related fused heterocyclic derivatives have great importance in the medicinal field as biological agents such as antimicrobial, anti-inflammatory, and anticancer agents [1–6]. The importance of pyrazole and pyrazolopyrimidine in the pharmacological industry as antitumor agents [7, 8] promoted us to synthesize new derivatives that may serve as new chemotherapeutic drugs.

**Fig. 1:**
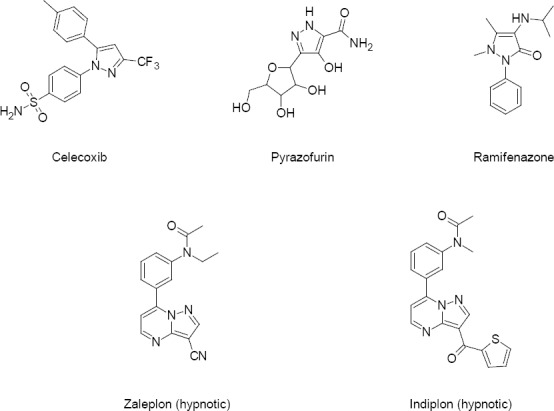
The structures of some drugs bearing the pyrazole and pyrazolopyrimidine moiety

A literature survey revealed that some drugs bearing pyrazole and pyrazolopyrimidine moieties are considered as the most active in drug manufacture such as *Celecoxib* [9]*, Pyrazofurin* [10], *Ramifenazone* [11], *Zaleplon* [12], and *Indiplon* [13], respectively [Fig. 1].

In light of these facts and as a continuation of our previous work in the synthesis of novel compounds with promising biological applications [14–16], we report herein the synthesis of new 5-aminopyrazole and pyrazolo[1,5-*a*]pyrimidine derivatives and an examination of their cytotoxic activity. The structures of the synthesized compounds were established based on elemental analysis and spectral data (IR, MS, ^1^H-NMR, and ^13^C-NMR).

## Results and Discussion

### Chemistry

*N*-substituted cyanoacetamide derivatives **1a–c** [17] were utilized as key starting materials in the synthesis of novel heterocyclic compounds (Schemes 1, 3, and 4). Cyanoacetamide derivatives **1a–c** were reacted with 4-methoxyphenylisothiocyanate in absolute ethanol in the presence of an equimolar amount of potassium hydroxide to give the corresponding intermediates (**2a–c**); when the latter was alkylated with methyl iodide in ethanol, it afforded the novel ketene *N,S*-acetals **3a–c**. The structures of **3a–c** were established on the basis of their elemental analysis and spectral data (MS, IR, ^1^H–NMR, and ^13^C-NMR). As an example, the mass spectrum of compound **3b** (*m/z* 353) [M^+^] revealed the molecular formula C_19_H_19_N_3_O_2_S. Its IR spectrum (KBr/cm^−1^) showed a band at 3357 corresponding to an NH group, a band at 2189 for a C≡N group, and a band at 1629 for a C=O group. Its ^1^H-NMR spectrum (DMSO-*d_6_*, δ ppm) revealed three singlets at 2.17, 2.21, and 3.72 representing -SCH_3_, -CH_3,_ and -OCH_3_ protons, respectively, four doublets at 6.92, 7.05, 7.25, and 7.37 corresponding to the aromatic protons (AB system, each with *J_HH_*=8.4 Hz), and two singlets at 9.37 and 11.82 assigned to two NH groups which were D_2_O exchangeable. Its ^13^C-NMR spectrum (DMSO-*d_6_*, δ ppm) was characterized by signals at 17.0, 119.2, and 165.3 assigned to -SCH_3_, -C≡N, and C=O carbons, respectively. The reaction of the compounds **3a–c** with hydrazine hydrate in refluxing ethanol gave the corresponding 5-amino-*N*-aryl-1*H*-pyrazole-4-carboxamides **4a–c** (Scheme 1). The structures of **4a–c** were established on the basis of their elemental analysis and spectral data (MS, IR, ^1^H–NMR, and ^13^C-NMR). Structure **4c** was supported by its mass spectrum (*m/z* 358) [M^+^], which agrees with its molecular formula C_17_H_16_ClN_5_O_2_. Its IR spectrum (KBr/cm^−1^) showed a band at 3347 and 3031 corresponding to NH and NH_2_ groups and a band at 1642 for a C=O group. Its ^1^H-NMR spectrum (DMSO-*d_6_*, δ ppm) displayed a singlet at 3.64 representing -OCH_3_ protons, a broad signal at 6.00 corresponding to the NH_2_ group which was D_2_O exchangeable, a multiplet at 6.77–7.51 related to the aromatic protons, and another three singlets at 8.30, 8.82, and 11.20 assignable to the three NH groups which were D_2_O exchangeable. Its ^13^C-NMR spectrum (DMSO-*d_6_*, δ ppm) was characterized by signals at 55.7, 87.9, and 163.6 assigned to -OCH_3_, C_4_ of the pyrazole moiety, and C=O carbons, respectively.

**Sch. 1:**
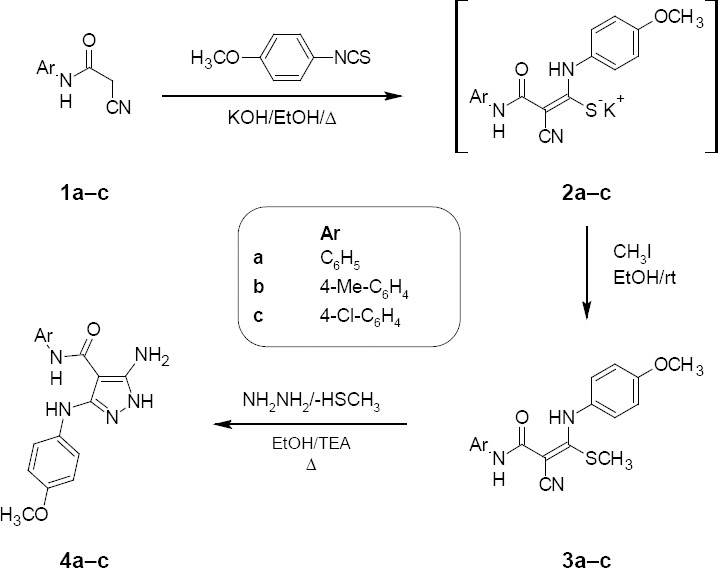
Synthesis of *N*-(aryl)-2-cyano-3-(4-methoxyphenylamino)-3-(methylthio)acrylamides 3a–c and 5-amino-*N*-aryl-3-(4-methoxyphenylamino)-1*H*-pyrazole-4-carboxamides 4a–c

**Sch. 2:**
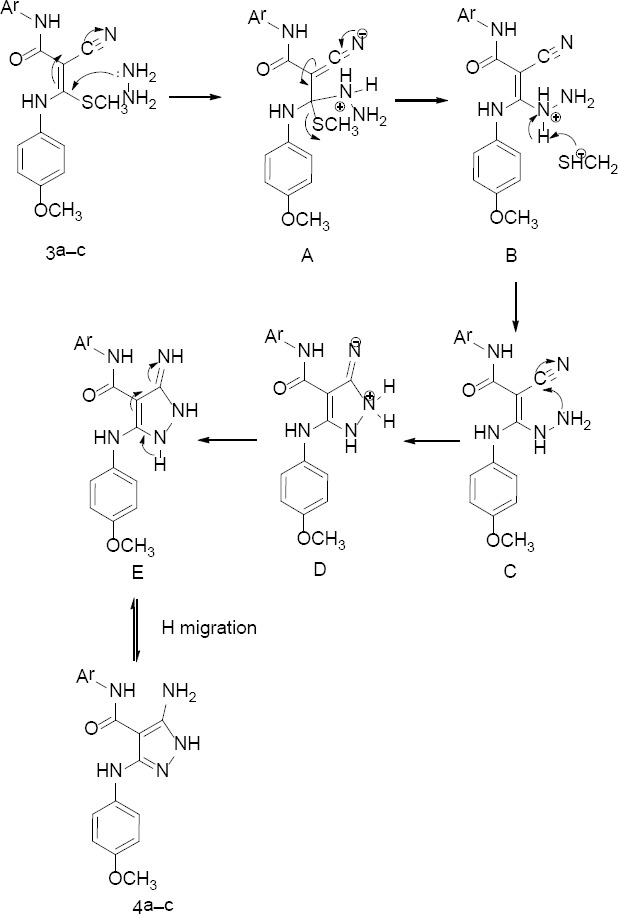
Mechanism for the formation of 5-amino-*N*-aryl-3-[(4-methoxyphenyl)amino]-1*H*-pyrazole-4-carboxamides 4a–c

**Sch. 3:**
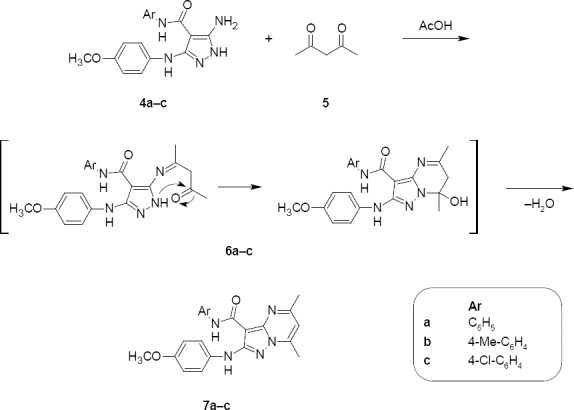
Synthesis of *N*-aryl-2-[(4-methoxyphenyl)amino]-5,7-dimethylpyrazolo[1,5-*a*]pyrimidine-3-carboxamides 7a–c

**Sch. 4:**
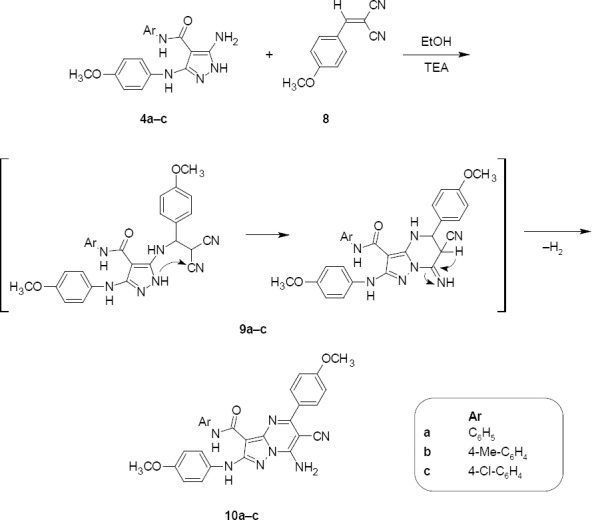
Synthesis of 7-amino-*N*-aryl-6-cyano-5-(4-methoxyphenyl)-2-[(4-methoxyphenyl)amino]pyrazolo[1,5-*a*]pyrimidine-3-carboxamides 10a–c

The possible formation of 5-aminopyrazoles **4a–c** is shown in Scheme 2. First, Michael addition to ketene *N,S*-acetals **3a–c** occurs with the lone pair of the NH_2_ group in hydrazine to form intermediate adduct **A**. Then, the methylthio ion is removed, which results in the formation of intermediate **B**. This methylthio ion abstracts the proton of the ammonium ion to produce an intermediate **C**. Subsequently, intermolecular cyclization occurs by the lone pair on the NH_2_ group, attacking the cyano group to produce intermediate **D**. The ammonium proton abstraction in **D** occurs to form **E**, followed by aromatic-driven 1,5-hydrogen migration to yield products **4a–c** [18].

Compounds **4a–c** were reacted with acetylacetone **5** in boiling glacial acetic acid to afford the corresponding new pyrazolo[1,5-*a*]pyrimidines **7a–c**. The formation of compounds **7a–c** was therefore assumed to proceed *via* an initial attack of the exocyclic amino group of **4a–c** on the keto group of the 1,3-dicarbonyl compound **5**, followed by intramolecular cyclization *via* the elimination of water (Scheme 3). The structures of **7a–c** were established on the basis of their elemental analysis and spectral data (MS, IR, ^1^H–NMR, and ^13^C-NMR). Structure **7c** was supported by its mass spectrum (*m/z* 421) [M^+^], which agrees with its molecular formula C_22_H_20_ClN_5_O_2_. Its IR spectrum (KBr/cm^−1^) showed a band at 3314 corresponding to two NH groups and a band at 1664 for a C=O group. Its ^1^H-NMR spectrum displayed three singlets at 2.55, 2.62, and 3.70 due to two -CH_3_ and -OCH_3_ protons, respectively, a signal at 6.92 corresponding to the H-6 proton of the pyrimidine nucleus, two doublets at 6.88 and 7.36 related to the four aromatic protons (AB system, *J_HH_*=7.8 Hz), another two doublets at 7.61 and 7.68 corresponding to the four aromatic protons in the other ring (AB system, *J_HH_*=6.7 Hz), and two singlets at 9.06 and 10.03 assignable to the two NH groups which were D_2_O exchangeable. Its ^13^C-NMR spectrum (CDCl_3_, δ ppm) was characterized by signals at 17.3, 24.6, 55.6, 108.5, and 163.0 assigned to two -CH_3,_ -OCH_3_, C_6_ of the pyrazolopyrimidine moiety and C=O carbons, respectively.

2-(4-Methoxybenzylidene)malononitrile **8** was reacted with **4a–c** in refluxing ethanol in the presence of triethylamine to give 7-amino-6-cyano-5-aryl-2-(arylamino)pyrazolo[1,5-*a*]pyrimidine-3-carboxamides **10a–c**. The formation of compounds **10a-c** is assumed to proceed *via* an initial attack of the exocyclic amino function of compounds **4a–c** on the *α,β*-unsaturated system in compound **8** followed by the intramolecular cyclization and spontaneous autooxidation through the loss of a hydrogen molecule [19] (Scheme 4). The structures of **10a–c** were established on the basis of their elemental analysis and spectral data (MS, IR, ^1^H–NMR, and ^13^C-NMR). As an example, structure **10b** was supported by its mass spectrum (*m/z* 520) [M^+^+1], which agrees with its molecular formula C_29_H_25_N_7_O_3_. Its IR spectrum (KBr/cm^−1^) showed an absorption band at 3447, 3296 which corresponds to the NH and NH_2_ groups, a band at 2211 due to C≡N, and a band at 1653 due to C=O. Its ^1^H-NMR spectrum displayed three singlets at 2.24, 3.71, and 3.84 due to -CH_3_ and two -OCH_3_ protons, respectively, six doublets at 6.85, 7.13, 7.16, 7.46, 7.51, and 7.97 related to the aromatic protons (AB system, *J_HH_*=9.2, 7.5, 8.5, 8.4, 8.2, and 8.0 Hz, respectively), and three singlets at 8.70, 9.78, and 12.76 assignable to NH_2_ and two NH groups, respectively, which were D_2_O exchangeable. Its ^13^C-NMR spectrum (CDCl_3_, δ ppm) was characterized by signals at 55.4, 55.6, 114.8, and 163.6 assigned to two -OCH_3_, -C≡N, and C=O carbons, respectively.

### Biological Evaluation

#### In Vitro Cytotoxic Screening

In the present study, some of the newly synthesized compounds were evaluated *in vitro* for their cytotoxic activities against the Ehrlich Ascites Carcinoma cells (EAC) where Doxorubicin was used as a standard drug. The results were expressed as the IC_50_ value, which corresponds to the concentration required for 50% inhibition of cell growth of the treated cells when compared to that of the control cells. From the results in Table 1, it was found that compounds **7a** (IC_50_=10 µg/ml) and **10c** (IC_50_=25 µg/ml) exhibited the highest cytotoxic activity compared to the reference drug, Doxorubicin (IC_50_=37.4 µg/ml). The compounds **4b** (IC_50_=50 µg/ml), **7b** (IC_50_=47µg/ml), and **7c** (IC_50_=42 µg/ml) displayed moderate cytotoxic activity against EAC cells, while compounds **4a**, **4c**, and **10b** showed lower activity than the reference drug.

**Tab. 1:**
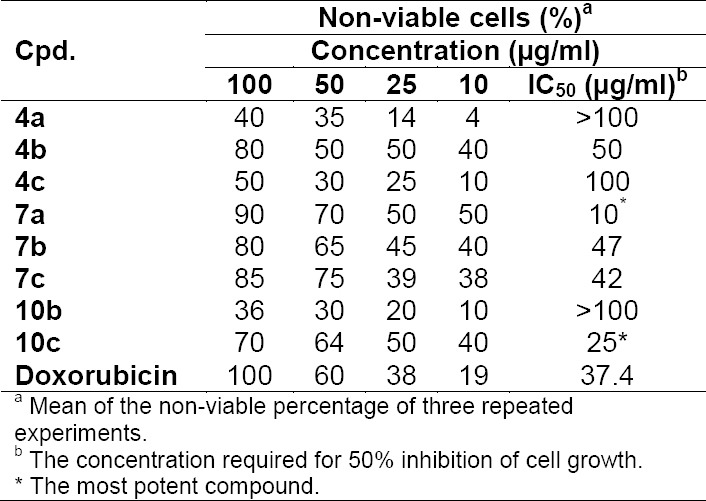
Cytotoxicity of the tested compounds against Ehrlich Ascites Carcinoma (EAC) cells

## Experimental

All melting points were measured on a Gallenkamp melting point apparatus and are uncorrected. The IR spectra were recorded (KBr disk) on a Perkin Elmer 1650 FTIR instrument. The ^1^H-NMR (500 MHz) and ^13^C-NMR (125 MHz) spectra were recorded on a Varian spectrometer using DMSO-*d*_6_ or CDCl_3_ as the solvent and TMS as an internal standard. Chemical shifts are reported in ppm. Mass spectra were recorded on a Varian MAT 112 spectrometer at 70 eV. Elemental analyses were performed at the Microanalytical Center, Cairo University, Egypt.

The progress of the reactions was monitored by thin-layer chromatography (TLC) using aluminum sheets coated with silica gel F_254_ (Merck) with viewing under short-wavelength UV lamp detection. All evaporations were carried out under reduced pressure at 40°C.

Reagents and solvents used in the synthesis were purchased from Sigma-Aldrich. Compounds **1a–c** were prepared according to the reported procedure [17].

### N-(Aryl)-2-cyano-3-[(4-methoxyphenyl)amino]-3-(methylthio)acrylamides (3a–c)

A mixture of *N*-substituted cyanoacetamide derivatives **1a-c** (0.01 mol) and 4-methoxyphenylisothiocyanate (0.01 mol) was heated for 5-10 min in ethanol (25 ml) containing potassium hydroxide (0.01 mol). After cooling, methyl iodide (0.01 mol) was added. The reaction mixture was stirred at room temperature for 1 h and then poured onto ice water. The precipitated product was filtered and recrystallized from ethanol.

#### 2-Cyano-3-[(4-methoxyphenyl)amino]-3-(methylthio)-N-phenylacrylamide (3a)

Light yellow prisms, m.p. 102–104°C, yield (71%). IR (KBr) ν_max_/cm^−1^ 3382 (NH), 2186 (C≡N), 1633 (C=O). ^1^H-NMR (DMSO-*d_6_*, δ ppm) 2.18 (s, 3H, SCH_3_), 3.72 (s, 3H, OCH_3_), 6.91 (d, 2H, aromatic, *J_HH_*=9.2 Hz), 7.02 (t, 1H, aromatic, *J_HH_*=7.7 Hz), 7.23 (t, 2H, aromatic, *J_HH_*=8.4 Hz), 7.25 (d, 2H, aromatic, *J_HH_*=8.4 Hz), 7.48 (d, 2H, aromatic, *J_HH_*=7.7 Hz), 9.46 (s, 1H, NH, D_2_O exchangeable), 11.78 (s, 1H, NH, D_2_O exchangeable). Anal. Calcd. (%) for C_18_H_17_N_3_O_2_S (339.41): C, 63.70; H, 5.05; N, 12.38. Found: C, 63.50; H, 5.20; N, 12.20%.

#### 2-Cyano-3-[(4-methoxyphenyl)amino]-N-(4-methylphenyl)-3-(methylthio)acrylamide (3b)

Colorless prisms, m.p. 175–176°C, yield (76%). IR (KBr) ν_max_/cm^−1^ 3357 (NH), 2189 (C≡N), 1629 (C=O). ^1^H-NMR (DMSO-*d_6_*, δ ppm) 2.17 (s, 3H, SCH_3_), 2.21 (s, 3H, CH_3_), 3.72 (s, 3H, OCH_3_), 6.92 (d, 2H, aromatic, *J_HH_*=8.4 Hz), 7.05 (d, 2H, aromatic, *J_HH_*=8.4 Hz), 7.25 (d, 2H, aromatic, *J_HH_*=8.4 Hz), 7.37 (d, 2H, aromatic, *J_HH_*=8.4 Hz), 9.37 (s, 1H, NH, D_2_O exchangeable), 11.82 (s, 1H, NH, D_2_O exchangeable). ^13^C-NMR (DMSO-*d*_6_, δ ppm) 17.0 (-SCH_3_), 20.9 (-CH_3_), 55.8 (-OCH_3_), 78.6 (C_2_, acrylamide), 114.9 (2C, aromatic), 119.2 (C≡N), 121.8, 126.3, 129.3, 131.5, 133.4, 136.1, 158.1 (10C, aromatic), 165.3 (C=O), 168.1 (C_3_, acrylamide). MS *m/z*: 353 [M^+^]. Anal. Calcd. (%) for C_19_H_19_N_3_O_2_S (353.44): C, 64.57; H, 5.42; N, 11.89. Found: C, 64.40; H, 5.64; N, 12.00%.

#### N-(4-Chlorophenyl)-2-cyano-3-[(4-methoxyphenyl)amino]-3-(methylthio)acrylamide (3c)

White crystals, m.p. 156–158°C, yield (70%). IR (KBr) ν_max_/cm^−1^ 3310 (NH), 2193 (C≡N), 1635 (C=O). ^1^H-NMR (DMSO-*d*_6,_ δ ppm) 2.19 (s, 3H, SCH_3_), 3.71 (s, 3H, OCH_3_), 6.91 (d, 2H, aromatic, *J_HH_*=6.9 Hz), 7.25 (d, 2H, aromatic, *J_HH_*=6.9 Hz), 7.30 (d, 2H, aromatic, *J_HH_*=6.9 Hz), 7.52 (d, 2H, aromatic, *J_HH_*=6.9 Hz), 9.62 (s, 1H, NH, D_2_O exchangeable), 11.68 (s, 1H, NH, D_2_O exchangeable). Anal. Calcd. (%) for C_18_H_16_ClN_3_O_2_S (373.86): C, 57.83; H, 4.31; N, 11.24. Found: C, 58.00; H, 4.10; N, 11.00%.

### 5-Amino-N-aryl-3-[(4-methoxyphenyl)amino]-1H-pyrazole-4-carboxamides (4a–c)

A mixture of compounds **3a–c** (0.01 mol), hydrazine hydrate (0.01 mol), and few drops of triethylamine in ethanol (30 ml) was refluxed for 4 h and then the solvent evaporated under reduced pressure. The resulting solid product was collected by filtration and recrystallized from ethanol.

#### 5-Amino-3-[(4-methoxyphenyl)amino]-N-phenyl-1H-pyrazole-4-carboxamide (4a)

White crystals, m.p. 175–177°C, yield (79%). IR (KBr) ν_max_/cm^−1^ 3354, 3030 (NH, NH_2_), 1645 (C=O). ^1^H-NMR (DMSO-*d*_6,_ δ ppm) 3.63 (s, 3H, OCH_3_), 5.98 (s, 2H, NH_2_, D_2_O exchangeable), 6.76 (d, 2H, aromatic, *J_HH_*=7.7 Hz), 6.98 (t, 1H, aromatic, *J_HH_*=7.7 Hz), 7.18 (t, 2H, aromatic, *J_HH_*=8.4 Hz), 7.25 (d, 2H, aromatic, *J_HH_*=7.7 Hz), 7.45 (d, 2H, aromatic, *J_HH_*=7.7 Hz), 8.30 (s, 1H, NH, D_2_O exchangeable), 8.74 (s, 1H, NH, D_2_O exchangeable), 11.22 (s, 1H, NH, D_2_O exchangeable). ^13^C-NMR (DMSO-*d*_6_, δ ppm) 55.6 (-OCH_3_), 88.3 (C_4_, pyrazole), 114.7, 117.5, 120.3, 123.3, 129.1, 137.6, 139.4 (11C, aromatic), 149.1 (C_5_, pyrazole), 150.9 (C_3_, pyrazole), 153.1 (C, aromatic), 163.6 (C=O). Anal. Calcd. (%) for C_17_H_17_N_5_O_2_ (323.35): C, 63.15; H, 5.30; N, 21.66. Found: C, 63.35; H, 5.15; N, 21.50%.

#### 5-Amino-3-[(4-methoxyphenyl)amino]-N-(4-methylphenyl)-1H-pyrazole-4-carboxamide (4b)

White crystals, m.p. 198–200°C, yield (82%). IR (KBr) ν_max_/cm^−1^ 3358, 3047 (NH, NH_2_), 1640 (C=O). ^1^H-NMR (DMSO-*d*_6,_ δ ppm) 2.21 (s, 3H, CH_3_), 3.64 (s, 3H, OCH_3_), 5.95 (s, 2H, NH_2_, D_2_O exchangeable), 6.76 (d, 2H, aromatic, *J_HH_*=8.4 Hz), 7.04 (d, 2H, aromatic, *J_HH_*=8.4 Hz), 7.16 (d, 2H, aromatic, *J_HH_*=8.4 Hz), 7.33 (d, 2H, aromatic, *J_HH_*=8.4 Hz), 8.30 (s, 1H, NH, D_2_O exchangeable), 8.66 (s, 1H, NH, D_2_O exchangeable), 11.21 (s, 1H, NH, D_2_O exchangeable). Anal. Calcd. (%) for C_18_H_19_N_5_O_2_ (337.38): C, 64.08; H, 5.68; N, 20.76. Found: C, 64.25; H, 5.50; N, 20.90%.

#### 5-Amino-N-(4-chlorophenyl)-3-[(4-methoxyphenyl)amino]-1H-pyrazole-4-carboxamide (4c)

White crystals, m.p. 190–192°C, yield (80%). IR (KBr) ν_max_/cm^−1^ 3347, 3031 (NH, NH_2_), 1642 (C=O). ^1^H-NMR (DMSO-*d*_6,_ δ ppm) 3.64 (s, 3H, OCH_3_), 6.00 (s, 2H, NH_2_, D_2_O exchangeable), 6.77–7.51 (m, 8H, aromatic), 8.30 (s, 1H, NH, D_2_O exchangeable), 8.82 (s, 1H, NH, D_2_O exchangeable), 11.20 (s, 1H, NH, D_2_O exchangeable). ^13^C-NMR (DMSO-*d*_6_, δ ppm) 55.7 (-OCH_3_), 87.9 (C_4_, pyrazole), 114.7, 117.7, 121.9, 126.8, 128.9, 137.4, 138.4 (11C, aromatic), 149.1 (C_5_, pyrazole), 151.1 (C_3_, pyrazole), 153.2 (C, aromatic), 163.6 (C=O). MS *m/z*: 358 [M^+^]. Anal. Calcd. (%) for C_17_H_16_ClN_5_O_2_ (357.79): C, 57.07; H, 4.51; N, 19.57. Found: C, 57.30; H, 4.40; N, 19.75%.

### Synthesis of N-Aryl-2-[(4-methoxyphenyl)amino]-5,7-dimethylpyrazolo[1,5-a]pyrimidine-3-carboxamide (7a–c)

A mixture of compounds **4a–c** (0.01 mol) with acetylacetone **5** (0.01 mol) in glacial acetic acid (20 ml) was refluxed for 6 h, then poured onto crushed ice, and the separated solid was filtered, dried well, and recrystallized from ethanol to afford compounds **7a–c**.

#### 2-[(4-Methoxyphenyl)amino]-5,7-dimethyl-N-phenylpyrazolo[1,5-a]pyrimidine-3-carboxamide (7a)

White crystals, m.p. 215–217°C, yield (85%). IR (KBr) ν_max_/cm^−1^ 3305 (NH), 1653 (C=O). ^1^H-NMR (DMSO-*d*_6,_ δ ppm) 2.50 (s, 3H, CH_3_), 2.56 (s, 3H, CH_3_), 3.70 (s, 3H, OCH_3_), 6.85 (d, 2H, aromatic, *J_HH_*=6.7 Hz), 6.89 (s, 1H, pyrimidine H-6), 7.06 (t, 1H, aromatic, *J_HH_=*7.4 Hz), 7.33 (t, 2H, aromatic, *J_HH_*=7.8 Hz), 7.59 (d, 2H, aromatic, *J_HH_*=8.8 Hz), 7.63 (d, 2H, aromatic, *J_HH_*=*7.8* Hz), 9.07 (s, 1H, NH, D_2_O exchangeable), 9.94 (s, 1H, NH, D_2_O exchangeable). MS *m/z*: 387 [M^+^]. Anal. Calcd. (%) for C_22_H_21_N_5_O_2_ (387.43): C, 68.20; H, 5.46; N, 18.08. Found: C, 68.28; H, 5.40; N, 18.00%.

#### 2-[(4-Methoxyphenyl)amino]-5,7-dimethyl-N-(4-methylphenyl)pyrazolo[1,5-a]pyrimidine-3-carboxamide (7b)

White crystals, m.p. 260–261°C, yield (88%). IR (KBr) ν_max_/cm^−1^ 3317 (NH), 1663 (C=O). ^1^H-NMR (DMSO-*d*_6,_ δ ppm) 2.25 (s, 3H, CH_3_), 2.61 (s, 3H, CH_3_), 2.69 (s, 3H, CH_3_), 3.70 (s, 3H, OCH_3_), 6.90 (d, 2H, aromatic, *J_HH_*=8.4 Hz), 7.00 (s, 1H, pyrimidine H-6), 7.16 (d, 2H, aromatic, *J_HH_*=8.3 Hz), 7.59 (d, 2H, aromatic, *J_HH_*=6.3 Hz), 7.66 (d, 2H, aromatic, *J_HH_*=6.3 Hz), 9.28 (s, 1H, NH, D_2_O exchangeable), 10.01 (s, 1H, NH, D_2_O exchangeable). MS *m/z*: 401 [M^+^]. Anal. Calcd. (%) for C_23_H_23_N_5_O_2_ (401.46): C, 68.81; H, 5.77; N, 17.44. Found: C, 68.75; H, 5.81; N, 17.50%.

#### N-(4-Chlorophenyl)-2-[(4-methoxyphenyl)amino]-5,7-dimethylpyrazolo[1,5-a]pyrimidine-3-carboxamide (7c)

White crystals, m.p. 254–256°C, yield (83%). IR (KBr) ν_max_/cm^−1^ 3314 (NH), 1664 (C=O). ^1^H-NMR (DMSO-*d*_6,_ δ ppm) 2.55 (s, 3H, CH_3_), 2.62 (s, 3H, CH_3_), 3.70 (s, 3H, OCH_3_), 6.88 (d, 2H, aromatic, *J_HH_*=7.8 Hz), 6.92 (s, 1H, pyrimidine H-6), 7.36 (d, 2H, aromatic, *J_HH_*=7.8 Hz), 7.61 (d, 2H, aromatic, *J_HH_*=6.7 Hz), 7.68 (d, 2H, aromatic, *J_HH_*=6.7 Hz), 9.06 (s, 1H, NH, D_2_O exchangeable), 10.03 (s, 1H, NH, D_2_O exchangeable). ^13^C-NMR (CDCl_3_, δ ppm) 17.3, 24.6 (-2CH_3_), 55.6 (-OCH_3_), 86.7 (C_3_, pyrazolopyrimidine), 108.5 (C_6_, pyrazolopyrimidine), 114.2, 118.7, 120.5, 128.0 (8C, aromatic), 134.0 (C_3a_, pyrazolopyrimidine), 137.5, 145.7, 146.0 (3C, aromatic), 154.2 (C_7_, pyrazolopyrimidine), 156.8 (C, aromatic), 156.9 (C_2_, pyrazolopyrimidine), 160.1 (C_5_, pyrazolopyrimidine), 163.0 (C=O). MS *m/z*: 421 [M^+^]. Anal. Calcd. (%) for C_22_H_20_ClN_5_O_2_ (421.88): C, 62.63; H, 4.78; N, 16.60. Found: C, 62.70; H, 4.75; N, 16.50%.

### Synthesis of 7-amino-N-aryl-6-cyano-5-(4-methoxyphenyl)-2-[(4-methoxyphenyl)amino]pyrazolo[1,5-a]pyrimidine-3-carboxamides (10a–c)

A mixture of compounds **4a-c** (0.01 mol) with 2-(4-methoxybenzylidene)malononitrile **8** (0.01 mol) and a catalytic amount of triethylamine (four drops) in absolute ethanol (30 ml) was refluxed for 6 h. The solvent was concentrated under reduced pressure and the solid obtained was collected and recrystallized from ethanol to give **10a-c**.

#### 7-Amino-6-cyano-5-(4-methoxyphenyl)-2-[(4-methoxyphenyl)amino]-N-phenyl-pyrazolo[1,5-a]pyrimidine-3-carboxamide (10a)

Yellow crystals, m.p. > 300ºC, yield (83%). IR (KBr) ν_max_/cm^−1^ 3440, 3356 (NH, NH_2_), 2210 (C≡N), 1650 (C=O). ^1^H-NMR (DMSO-*d*_6,_ δ ppm) 3.71 (s, 3H, OCH_3_), 3.85 (s, 3H, OCH_3_), 6.86 (d, 2H, aromatic, *J_HH_*=8.9 Hz), 7.06 (t, 1H, aromatic, *J_HH_*=8.6 Hz), 7.15 (d, 2H, aromatic, *J_HH_*=8.6 Hz), 7.34 (t, 2H, aromatic, *J_HH_*=7.7 Hz), 7.58 (d, 2H, aromatic, *J_HH_*=7.9 Hz), 7.82 (d, 2H, aromatic, *J_HH_*=8.9 Hz), 7.95 (d, 2H, aromatic, *J_HH_*=8.8 Hz), 9.02 (s, 2H, NH_2_, D_2_O exchangeable), 9.21 (s, 1H, NH, D_2_O exchangeable), 10.12 (s, 1H, NH, D_2_O exchangeable). Anal. Calcd. (%) for C_28_H_23_N_7_O_3_ (505.53): C, 66.52; H, 4.59; N, 19.39. Found: C, 66.60; H, 4.55; N, 19.45%.

#### 7-Amino-6-cyano-5-(4-methoxyphenyl)-2-[(4-methoxyphenyl)amino]-N-(4-methylphenyl)-pyrazolo[1,5-a]pyrimidine-3-carboxamide (10b)

Yellow crystals, m.p. 248–250°C, yield (78%). IR (KBr) ν_max_/cm^−1^ 3447, 3296 (NH, NH_2_), 2211 (C≡N), 1653 (C=O). ^1^H-NMR (DMSO-*d*_6,_ δ ppm) 2.24 (s, 3H, CH_3_), 3.71 (s, 3H, OCH_3_), 3.84 (s, 3H, OCH_3_), 6.85 (d, 2H, aromatic, *J_HH_*=9.2 Hz), 7.13 (d, 2H, aromatic, *J_HH_*=7.5 Hz), 7.16 (d, 2H, aromatic, *J_HH_*=8.5 Hz), 7.46 (d, 2H, aromatic, *J_HH_*=8.4 Hz), 7.51 (d, 2H, aromatic, *J_HH_*=8.2 Hz), 7.97 (d, 2H, aromatic, *J_HH_*=8.0 Hz), 8.70 (s, 2H, NH_2_, D_2_O exchangeable), 9.78 (s, 1H, NH, D_2_O exchangeable), 12.76 (s, 1H, NH, D_2_O exchangeable). ^13^C-NMR (CDCl_3_, δ ppm) 21.0 (-CH_3_), 55.4, 55.6 (-2OCH_3_), 77.0 (C_6_, pyrazolopyrimidine merged with the peak of CDCl_3_), 92.2 (C_3_, pyrazolopyrimidine), 114.4, 144.6 (4C, aromatic), 114.8 (C≡N), 119.6, 121.9, 127.8, 129.3, 129.6 (9C, aromatic), 131.5 (C_3a_, pyrazolopyrimidine), 132.1, 133.1, 136.4, 150.6 (4C, aromatic), 156.1 (C_2_, pyrazolopyrimidine), 162.3 (C, aromatic), 163.2 (C_5_, pyrazolopyrimidine), 163.6 (C=O), 167.4 (C_7_, pyrazolopyrimidine). MS *m/z*: 520 [M^+^+1]. Anal. Calcd. (%) for C_29_H_25_N_7_O_3_ (519.55): C, 67.04; H, 4.85; N, 18.87. Found: C, 67.15; H, 4.80; N, 18.90%.

#### 7-Amino-N-(4-chlorophenyl)-6-cyano-5-(4-methoxyphenyl)-2-[(4-methoxyphenyl)amino]pyrazolo[1,5-a]pyrimidine-3-carboxamide (10c)

Yellow crystals, m.p. 240–242°C, yield (80%). IR (KBr) ν_max_/cm^−1^ 3433, 3297 (NH, NH_2_), 2215 (C≡N), 1660 (C=O). ^1^H-NMR (DMSO-*d*_6,_ δ ppm) 3.71 (s, 3H, OCH_3_), 3.85 (s, 3H, OCH_3_), 6.86 (d, 2H, aromatic, *J_HH_*=9.0 Hz), 7.14 (d, 2H, aromatic, *J_HH_*=8.8 Hz), 7.39 (d, 2H, aromatic, *J_HH_*=6.7 Hz), 7.48 (d, 2H, aromatic, *J_HH_*=8.0 Hz), 7.63 (d, 2H, aromatic, *J_HH_*=8.6 Hz), 7.98 (d, 2H, aromatic, *J_HH_*=10.0 Hz), 8.63 (s, 2H, NH_2_, D_2_O exchangeable), 9.87 (s, 1H, NH, D_2_O exchangeable), 12.78 (s, 1H, NH, D_2_O exchangeable). Anal. Calcd. (%) for C_28_H_22_ClN_7_O_3_ (539.97): C, 62.28; H, 4.11; N, 18.16. Found: C, 62.20; H, 4.15; N, 18.20%.

### Biological Experiments

#### In Vitro Cytotoxic Activity

Doxoroubicin, the reference drug which was used in this study, is one of the most effective antitumor agents used to produce regressions in acute leukemia, Hodgkin disease, and other lymphoma. The relationship between the survival ratio and drug concentration was plotted to obtain the survival curve of the Ehrlich Ascites Carcinoma (EAC) cells. The parameter IC_50_ is the concentration of the drugs inducing 50% inhibition of cell growth of the treated cells in comparison with the growth of the control cells.

#### Procedure

The EAC cells were obtained by needle aspiration of the ascetic from preinoculated mice under aseptic conditions. The tumor cells suspension (2.5×10^6^ cells/ml) was prepared in RPMI-1640 media. The tested compounds were prepared with various dilutions by dissolving: 100, 50, 25, and 10 µg in DMSO (1 ml). In a set of sterile test tubes, to 0.8 ml of RPMI-1640 media containing (glutamine, fetal calf serum as the nutrient, streptomycin, and pencillin) 0.1 ml of each tested compound was added. The test tube was then incubated at 37°C for 2 h. The trypan blue exclusion test was carried out to calculate the percentage of non-viable cells after 2 h of incubation:


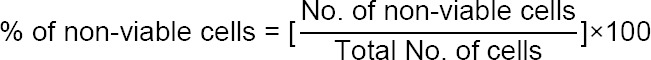


## Conclusion

In conclusion, *N*-substituted cyanoacetamide derivatives **1a-c** were used as starting materials for the synthesis of some new *N*-(aryl)-2-cyano-3-(methylthio)acrylamides **3a–c**, 5-amino-1*H*-pyrazoles **4a–c**, and pyrazolo[1,5-*a*]pyrimidines **7a–c** and **10a–c**. The new synthesized compounds were characterized by analytical and spectroscopic data. Some selected new compounds were screened for their potential cytotoxic activity. The results of the cytotoxicity for the tested compounds against Ehrlich Ascites Carcinoma (EAC) cells indicated that the pyrazolo[1,5-*a*]pyrimidine derivatives **7a** (IC_50_=10 µg/ml) and **10c** (IC_50_=25 µg/ml) were found to have the most potent growth inhibitory activity against EAC cells in comparison with the reference drug, Doxorubicin (IC_50_=37.4 µg/ml). Accordingly, this class of compounds could be considered as useful templates for future development, derivatization, or modification to obtain more potent and selective antitumor agents.
